# Radioguided Parathyroidectomy with Portable Mini Gamma-Camera for the Treatment of Primary Hyperparathyroidism

**DOI:** 10.1155/2015/134731

**Published:** 2015-09-15

**Authors:** Claudio Casella, Pierluigi Rossini, Carlo Cappelli, Chiara Nessi, Riccardo Nascimbeni, Nazario Portolani

**Affiliations:** ^1^Department of Molecular and Translational Medicine, Spedali Civili, 3rd Division of General Surgery, University of Brescia, 25123 Brescia, Italy; ^2^Nuclear Medicine, A. O. Carlo Poma, 46100 Mantova, Italy; ^3^Department of Medical and Surgical Sciences, Spedali Civili, 2nd Division of Internal Medicine, University of Brescia, 25123 Brescia, Italy

## Abstract

*Background*. A proper localisation of pathological parathyroid glands is essential for a minimally invasive approach in the surgical treatment of primary hyperparathyroidism (PHP). The recent introduction of portable mini gamma-cameras (pMGCs) enabled intraoperative scintigraphic scanning. The aim of our study is to evaluate the efficacy of this new method and compare it with the preoperative localisation surveys. *Methods*. 20 patients were studied; they were evaluated preoperatively by neck ultrasound and ^99mm^Tc-sestaMIBI-scintigraphy and intraoperatively with the pMGC IP Guardian 2. The results obtained from the three evaluations were compared. *Results*. The pMGC presented a sensitivity of 95%, a specificity of 98.89%, and a diagnostic accuracy of 98.18%, which were higher than those of preoperative ultrasound (sensitivity 55%; specificity 95%; diagnostic accuracy 87%) and scintigraphy with ^99mm^Tc-sestaMIBI (sensitivity 73.68%; specificity 96.05%; diagnostic accuracy 91.58%). *Conclusions*. The pMGC can be used effectively as an intraoperative method to find the correct location of the pathological parathyroid glands. The pMGC is more reliable than the currently used preoperative and intraoperative localisation techniques.

## 1. Introduction

PHP is an increasingly more frequent endocrine disorder, being third after diabetes and thyroid disease [1].

Surgical therapy is indicated in patients with symptomatic PHP [2] whereas guidelines for surgical indications in patients with asymptomatic PHP were recently published [3]. PHP treatment has developed greatly over time, going from bilateral cervical exploration to minimally invasive surgical techniques [4]. This was made possible by the introduction of, both preoperative and intraoperative, localisation techniques investigations that allow the precise identification of pathological parathyroid glands and the introduction of the intraoperative dosage of parathyroid hormone (ioPTH) [5, 6].

Ultrasound and scintigraphy with ^99m^Tc-sestaMIBI are most frequently used, but their sensitivity and specificity do not always enable the correct localisation of parathyroid lesions, especially in cases of multiglandular hyperplasia, or ectopic glands, or in patients with concomitant thyroid disease [7].

This fact led to the development of technologies or devices in order to localise lesions intraoperatively [8, 9]. The recent introduction of pMGCs enables intraoperative scintigraphic scanning of parathyroid glands [10, 11], being an alternative to other intraoperative investigations, such as radioguided surgery [8] or methylene blue [12], which have been used so far.

There are few reports in literature about the use of pMGC in PHP treatment [10, 11].

In our study, we evaluated the effectiveness of using the pMGC in a group of patients undergoing parathyroidectomy for PHP, comparing the results obtained by this method with the investigation of preoperative localisation (ultrasound and scintigraphy).

## 2. Materials and Methods

We analysed data from 20 patients undergoing parathyroid surgery for PHP in the period between January 2011 and April 2013.

Demographic features, clinical symptoms of PHP, and preoperative laboratory tests, with particular reference to serum calcium and parathyroid hormone (PTH) levels, were assessed in all these patients. The data obtained on preoperative imaging studies (ultrasound of the cervical region and scintigraphy with ^99m^Tc-sestaMIBI) were recorded in all patients.

All patients underwent surgery and in all cases the pMGC IP Guardian 2 (Li-Tech, Rome, Italy) was used.

The pMGC used in the study had the following technical features:

(i) Detection head of size 7 cm (*H*) × 7 cm (*L*) × 27 cm (*P*), detection area: 44.1 mm × 44.1 mm, and weight: 1.2 kg.
(ii) Crystal: Csl(T1) matrix 18 × 18 elements.
(iii) Collimator: tungsten, square holes, and length 24 mm.
(iv) Energy resolution: 20% at 141 keV (^99mm^Tc).
(v) Control unit: 10.4-inch screen (1924 × 768), Microsoft Windows XP Tablet PC Edition operating system; processor: Intel Centrino Mobile 2 MB L2, 1,20 GHz; size: 25.6 cm × 25.6 cm × 2.43 cm (depth); weight: 1.5 kg.

**Figure 1:**
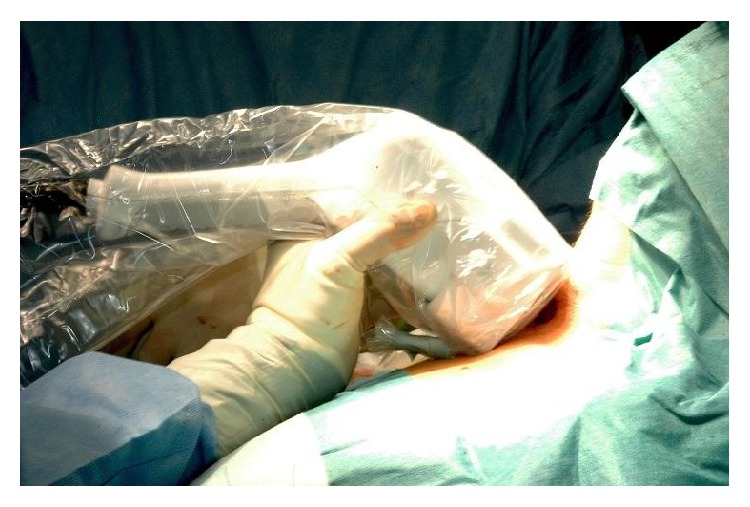
Intraoperative scans acquisition.

**Figure 2:**
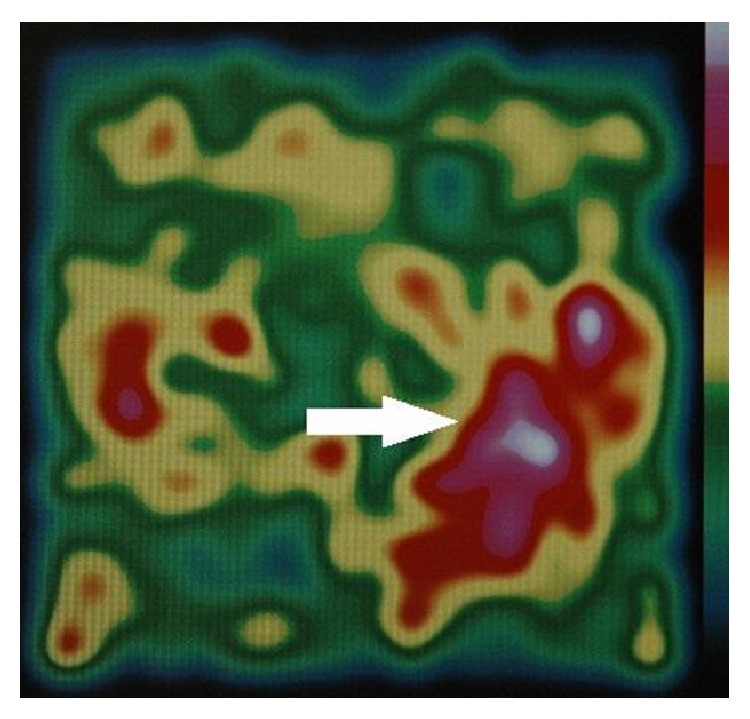
Lower left adenoma (white arrow).

According to the intraoperative image acquisition protocol, we intravenously infused 185 MBq of ^99m^Tc-sestaMIBI immediately after the induction of general anaesthesia. Subsequently, the patient was placed on the operating table and the operating field was prepared, so that enough time would pass for the isotope to concentrate in the pathological parathyroid tissue (about 15–20 minutes). Scintigraphic scans were then acquired, positioning the sensing head above the neck (Figures 1 and 2).

After proper localisation, a minimally invasive parathyroidectomy with minicervicotomy was started.

After removing the pathological gland, further images of the removed material and the operating field were acquired in order to confirm the radioactivity of the removed material and the absence of other sources of uptake in the neck.

The levels of ioPTH were recorded in all cases, according to Miami criteria [13].

We then compared the ability of the preoperative and intraoperative imaging techniques to correctly locate the parathyroid lesion not only in terms of right or left laterality, but also in terms of upper or lower quadrant with respect to thyroid isthmus. The results provided by the different methods of localisation were compared to the surgical and pathological findings.

We also recorded the operative times (from skin to skin) of the patients examined, comparing them with the operative time of a homogenous (observation period, number, age, sex, symptoms, preoperative investigations performed, and surgeon) group of patients previously treated surgically without using the pMGC.

In the long term, all patients were monitored with clinical outpatient visits and laboratory tests (PTH and serum calcium) 1, 6, and 12 months after surgery.

## 3. Results

Of 20 accrued patients, 13 (65%) were females and 7 (35%) were males. The average age was 63.7 years, with a range from 40 to 80 years (average: 65 years).

No patient was affected by or had a documented family history of multiple endocrine neoplasia (MEN 1 or MEN 2A).

Clinically 6 patients (30%) were PHP asymptomatic and 14 (70%) were PHP symptomatic (symptoms shown in Table 1).

None of the treated patients had a concomitant thyroid disease, and none had previously undergone surgery on the cervical region.

Preoperatively, all patients had hypercalcemia with an average serum calcium of 12.26 mg/dL (IC 95%: 11.49–13.03). All cases showed an elevated PTH with a mean value of 350.1 pg/mL (IC 95%: 233.59–466.61) (Table 1).

The data obtained from preoperative imaging studies (ultrasound and scintigraphy with ^99m^Tc-sestaMIBI), performed in all patients, are depicted in Table 2.

All patients underwent surgery, according to the indications provided by the latest available guidelines [3] for surgical treatment of patients with symptomatic or asymptomatic PHP.

All surgical findings were confirmed by frozen section examination at the time of surgery and by definitive histopathology of the excised tissue. The details of preoperative and intraoperative localisation data, as well as the surgical and pathologic findings, are given in Tables 2 and 3.

Having considered the results obtained with the ultrasound, the preoperative scintigraphy, and the pMGC, we evaluated the ability of the three methods to properly locate the pathological glands on the basis of the side and of the quadrant of the neck. As regards side localisation, neck ultrasound showed a sensitivity of 95% and a specificity of 100% with a diagnostic accuracy of 99%, a positive predictive value (PPV) of 100%, and a negative predictive value (NPV) of 98.76%. When considering quadrant localisation, the values decreased substantially with a sensitivity of 55%, a specificity of 95%, a diagnostic accuracy of 87%, PPV of 73.33%, and NPV of 89.41%.

The preoperative scintigraphy with ^99m^Tc-sestaMIBI showed sensitivity, specificity, diagnostic accuracy, PPV, and NPV results of 100% with regard to side localisation, whereas quadrant location showed values of 73.68% and 96.05%, respectively, for sensitivity and specificity, with a diagnostic accuracy of 91.58%, PPV of 82.35%, and NPV of 93.59%.

With side localisation the pMGC showed sensitivity, specificity, diagnostic accuracy, PPV, and NPV values of 100%, while with quadrant localisation it showed sensitivity and specificity levels of 95% and 98.89%, respectively, a diagnostic accuracy of 98.18%, PPV of 95%, and NPV of 98.88% (Tables 4 and 5).

In 7 cases (35%) the pMGC gave more accurate information than the neck ultrasound and in 5 cases (25%) more than the preoperative scintigraphic scans.

After removal of parathyroid lesions, MGC scanning demonstrated no sources of residual radioactivity in the operating field of any patient.

The ioPTH according to Miami criteria [13] showed a significant decrease (over 50%) in all patients (100%).

The average operative time was 37.75 minutes (IC 95%): 31.06–44.44 with a median time of 32 minutes. The comparison with a previous group of patients undergoing parathyroidectomy showed no significant differences in terms of average and median duration (39.2 minutes (IC 95%: 28.22–50.17) and 30 minutes, resp.).

There was no mortality or morbidity in the study group.

Postoperative 3rd-day monitoring of PTH and serum calcium demonstrated the following mean values: PTH 28.64 pg/mL (IC 95%: 22.48–38.4), with a decrease of 328.29 pg/mL from preoperative data, and serum calcium 9.22 mg/dL (IC 95%: 7.64–10.8) with a decrease of 3.69 mg/dL from preoperative data. At the clinical and laboratory follow-up after 1, 6, and 12 months, no persistent or recurrent PHP was detected.

## 4. Discussion

The traditional surgical approach to PHP treatment consisted of bilateral cervical exploration [14, 15]: this technique led to a success rate of up to 95%, when performed by dedicated surgeons [16]. As 85% of PHP cases are supported by a single parathyroid adenoma [17], minimally invasive surgery was introduced, with a focal approach to parathyroid lesions, made possible thanks to the improvements in preoperative localisation and to the introduction of ioPTH [4].

The most frequently used preoperative localisation methods are neck ultrasound and scintigraphy with ^99m^Tc-sestaMIBI [18]. The ultrasound has a sensitivity of 76.1% (range 30%–88%) [19, 20], which reaches a value of 97% [21], and a specificity of 40% [20] in case of typical cervical localisation.

The scintigraphy with ^99m^Tc-sestaMIBI provides information of functional type [22]. Its sensitivity for detecting single adenomas varies between 68 and 95%, which decreases to 44% in case of hyperplasia and 30% in case of double adenomas [18]. The SPECT (Single Photo Emission Computed Tomography) technique associated with CT (SPECT/CT) is useful in case of concomitant nodular thyroid disease or ectopic parathyroid glands (sensitivity over 90%) [23].

The CT and RMN are usually reserved for persistent or recurrent cases of PHP or ectopic adenomas localisation or when the ultrasound and the scintigraphy do not identify the same localisation [24, 25].

Intraoperative localisation techniques have been introduced in order to guide the surgeon during the parathyroidectomy, overcoming the limitations of preoperative imaging [12, 26].

In 1971 Dudley [12] was the first to report the intraoperative use of methylene blue: this method, however, has limitations, arising from the fact that even the normal parathyroid glands may be discoloured, just like thyroid nodules and lymph nodes [9].

The use of a gamma probe is the basis of minimally invasive radioguided parathyroidectomy (MIRP), proposed for the first time by Norman and Chheda in 1997 [26].

The use of gamma probes allows the detection of sources of radiotracers administered at the time of induction of anaesthesia [27].

Gamma probes, however, translate focus intensity into count rate and audio signalling and as such do not guarantee the more precise localisation given by imaging [28].

A further improvement was provided by the introduction of the pMGC, small in size and lightweight, which can provide scintigraphic images during surgery [10, 29, 30]. The use of the pMGC involves ^99m^Tc-sestaMIBI being injected before surgery and the acquisition of scans before the lesion is removed, in order to locate the lesion itself, and also after it has been removed, to confirm the excision of the whole pathological tissue, along with the scanning of the specimens [10]. The first clinical application of this method appeared in 2007 [10].

The pMGC presented high levels of sensitivity and specificity, higher than those of the preoperative ultrasound and scintigraphy [10, 11], and appears to be useful in cases of concomitant thyroid disease and in cases with negative preoperative studies [11].

Estrems and colleagues [11] evaluated the feasibility of this method in a group of 29 patients: side localisation with pMGC showed a sensitivity of 86.6% and a specificity of 90.9% compared to the 79.3% and 92.5%, respectively, of preoperative investigations (ultrasound + scintigraphy) while quadrant localisation showed a sensitivity of 83.3% and a specificity of 90.9%, when compared to 48.35% and 72.7%, respectively, reported in the preoperative surveys.

In our experience the pMGC properly localised all lesions by side (diagnostic accuracy 100%) with both a sensitivity and a specificity of 100%, while as far as quadrant was considered pMGC showed a diagnostic accuracy of 98.1%, a sensitivity of 95.0%, and a specificity of 98.8% (Tables 4 and 5).

In particular, its use guaranteed a more precise localisation of lesions in 7 cases (35%) with respect to ultrasound scan and in 5 cases (25%) with respect to scintigraphy with ^99m^Tc-sestaMIBI. The data obtained from our experience are comparable to those reported previously [10, 11, 31].

This technology appears to be a potential alternative to ioPTH measurement allowing obtaining and comparing easy-to-read images before and after excision of the parathyroid lesions [31, 32]. The absence of radiotracer uptake sources in the postexcision images confirms the completeness of the parathyroidectomy, completeness that is usually confirmed by the significant fall in PTH, also.

Despite the limited number of patients studied, our study has confirmed the possibility of replacing the ioPTH measurement with the intraoperative use of the pMGC, because in all cases the images obtained after removal of the 12 parathyroid lesions were comparable to the fall in ioPTH levels. The pMGC may also be a possible alternative to preoperative scintigraphy with ^99m^Tc-sestaMIBI.

As far as operative time is concerned, there was no statistical difference between the duration of the mini-invasive radioguided parathyroidectomy with pMGC and the duration of a parathyroidectomy guided only by preoperative studies (*p* > 0.05). This may lead to the inference that the additional time used to acquire the images was offset by the easier retrieval of surgical lesions, made possible by the more precise information provided by the pMGC.

## 5. Conclusions

We believe that the pMGC may be used as an intraoperative method to locate the correct position of the pathological parathyroid glands. The pMGC is more reliable than the preoperative and intraoperative localisation techniques used so far. Our study has also confirmed that the pMGC could replace the ioPTH measuring, by comparing the images obtained before and after the excision of the parathyroid lesions.

## Figures and Tables

**Table 1:** Patients with PHP.

Age	
Average (IC 95%)	63.7 (58.97–68.43)
Gender	
F	13 (65%)
M	7 (35%)
Serum calcium	
Average (IC 95%)	12.26 mg/dL (11.49–13.03)
PTH	
Average (IC 95%)	350.1 pg/mL (233.59–466.61)
Symptoms	
No	6 (30%)
Yes	14 (70%)
Kidney	7 (35%)
Osteoarticular	6 (30%)
Neuropsychic	2 (10%)
Digestive	1 (5%)
Acute hypercalcemia	1 (5%)

**Table 2:** Results of preoperative and intraoperative surgical findings and pathological anatomical study locators.

	Ultrasound	Scintigraphy	Portable MGC	Surgical findings	Anatomy-pathological findings
1	Left	Lower left	Lower left	Lower left	Lower left
2	Lower left	Lower left	Lower left	Lower left	Lower left
3	Lower left	Lower left	Lower left	Lower left	Lower left
4	Right	Middle right	Upper right	Upper right	Upper right
5	Middle left	Left	Upper left	Upper left	Upper left
6	No injuries	Lower left	Lower left	Lower left	Lower left
7	Lower right	Right	Lower right	Lower right	Lower right
8	Right	Paratracheal right	Paratracheal right	Paratracheal right	Paratracheal right
9	Middle left	Left	Middle left	Upper left	Upper left
10	Lower right	Lower right	Lower right	Lower right	Lower right
11	Lower right	Lower right	Lower right	Lower right	Lower right
12	Lower right	Lower right	Lower right	Lower right	Lower right
13	Middle right	Lower right	Lower right	Lower right	Lower right
14	Middle left	Lower left	Lower left	Lower left	Lower left
15	Upper left	Upper left	Upper left	Upper left	Upper left
16	Right	Upper left	Upper right	Upper right	Upper right
17	Lower left	Middle left	Lower left	Lower left	Lower left
18	Lower right	Lower right	Lower right	Lower right	Lower right
19	Upper left	Upper left	Upper left	Upper left	Upper left
20	Upper left	Middle left	Upper left	Upper left	Upper left

**Table 3:** Surgical findings.

Lower left adenoma	6 (30%)
Lower right adenoma	6 (30%)
Upper left adenoma	5 (25%)
Upper right adenoma	2 (10%)
Ectopic adenoma	1 (5%) *paratracheal right*

**Table 4:** Sensitivity, specificity, diagnostic accuracy, PPV, and NPV of the localisation investigations used according to the side of the neck.

	Sensitivity	Specificity	Diagnostic accuracy	PPV	NPV
Ultrasound	95%	100%	99%	100%	98.76%
Preoperative scintigraphy	100%	100%	100%	100%	100%
pMGC	100%	100%	100%	100%	100%

**Table 5:** Sensitivity, specificity, diagnostic accuracy, PPV, and NPV of the localisation used according to the quadrant of the neck.

	Sensitivity	Specificity	Diagnostic accuracy	PPV	NPV
Ultrasound	55%	95%	87%	73.33%	89.41%
Preoperative scintigraphy	73.68%	96.05%	91.58%	82.35%	93.59%
pMGC	95%	98.89%	98.18%	95%	98.88%
